# Diverse drug-resistant subpopulations of *Mycobacterium tuberculosis* are sustained in continuous culture

**DOI:** 10.1098/rsif.2016.0745

**Published:** 2016-11

**Authors:** Diepreye Ayabina, Charlotte Hendon-Dunn, Joanna Bacon, Caroline Colijn

**Affiliations:** 1 Department of Mathematics, Imperial College London, South Kensington Campus, London SW7 2AZ, UK; 2 Public Health England, National Infection Service, Porton Down, Salisbury SP4 0JG, UK

**Keywords:** *Mycobacterium tuberculosis*, diversity, resistance, competition

## Abstract

Drug resistance to tuberculosis (TB) has become more widespread over the past decade. As such, understanding the emergence and fitness of antibiotic-resistant subpopulations is crucial for the development of new interventions. Here we use a simple mathematical model to explain the differences in the response to isoniazid (INH) of *Mycobacterium tuberculosis* cells cultured under two growth rates in a chemostat. We obtain posterior distributions of model parameters consistent with data using a Markov chain Monte Carlo (MCMC) method. We explore the dynamics of diverse INH-resistant subpopulations consistent with these data in a multi-population model. We find that the simple model captures the qualitative behaviour of the cultures under both dilution rates and also present testable predictions about how diversity is maintained in such cultures.

## 1. Introduction

The battle to eradicate tuberculosis (TB), a disease caused by the pathogen *Mycobacterium tuberculosis*, has been on for more than half a century. In 2014, the World Health Organization estimated 9.6 million new TB cases and 1.5 million TB deaths [1]. The management of human TB relies heavily on vaccination, case finding and antibiotic treatment. Current treatments for TB are complex and lengthy leading to incomplete treatment, non-compliance and the development of multi-drug resistance (MDR). Unfortunately, on a global scale, drug-resistant (DR) forms of TB have become more widespread over the past decade, with an estimated 3.3% of new cases and 20% of previously treated cases with MDR-TB in 2015 [1]. In particular, isoniazid (INH) and rifampicin form the core of standard treatment regimens, and resistance to them is a keystone of MDR tuberculosis.

Understanding the emergence and fitness of antibiotic-resistant subpopulations is crucial for the development of new interventions. Because it is a highly clonal bacterium, *M. tuberculosis* acquires antibiotic resistance through mutation as opposed to DNA acquisition. For INH, clinically relevant resistance mutations have been identified, primarily in *katG* and *inhA*. katG encodes for the catalase-peroxidase responsible for activating the pro-drug INH and approximately 50–90% of all clinical INH-resistant isolates have a mutation at *katG* codon Ser315 [2]. In addition to *katG* and *inhA*, other mutated genes have been found in INH*^R^* (resistant) isolates [3–8]. Resistance can arise due to selection of randomly generated pre-existing resistance-conferring mutations, or by less direct mechanisms such as development of tolerance to hostile environments which can then confer compensatory advantages in the event that a population is exposed to antibiotics [9–11]. Drug tolerance may also be due to the expression of a phenotype that limits drug activity. For example, during INH exposure, epigenetic events leading to KatG pulsing result in a pattern of dynamic persistence [12]. Exposure to antibiotics can also lead to hypermutability and resistance. It has previously been shown upon INH treatment in guinea pigs that a slowing in the bactericidal activity of INH following an early rapid reduction in the bacterial population resulted from the selection of phenotypically tolerant slow-growing ‘persisters’ and not the emergence of resistant subpopulations [13]; these persisters may play an additional role in the evolution of resistance [14]. In contrast with these findings, hollow-fibre studies suggested that the cessation of the bactericidal activity of INH was as a result of a rapid emergence of antibiotic resistance and not the depletion of the exponential phase growth [15]. However, this requires further investigation as clinical data on the levels of antibiotic resistance in patients during early bactericidal activity studies revealed that antibiotic resistance was never encountered in INH monotherapy trials [16]. It remains unclear how the slow growth of *M. tuberculosis* contributes to INH tolerance. It is possible that slow-growing, metabolically inactive bacilli predominate in sputum after initial INH-mediated sterilization of the exponentially growing bacilli. One approach to answering these questions is to study the response of TB to different growth conditions in culture.

Bacilli in pulmonary cavities are thought to be growing in an aerobic environment and, therefore, behave in a way that can be imitated by *in vitro* systems like the chemostat [17]. The chemostat has been used to study and capture the response of MBT cells to specific challenges such as carbon limitation [18,19], oxygen limitation [20] and nutrient limitation [21]. Chemostat models of *M. tuberculosis* growing under fast and slow dilution are thought to be good models for the different phases of tuberculosis, an acute phase and an asymptomatic/persistent phase, respectively [19,22,23], although it is clear that an *in vitro* system cannot truly capture the bacteria's environment within a host. However, TB is challenging to work with, there are limited animal models, and the ability to control the conditions and intervene in a controlled way presents good opportunities to directly observe the evolution of TB under known selective pressures. Briefly, a chemostat consists of three compartments: the nutrient reservoir, the vessel containing the bacterial culture (growth chamber) and a tank to collect waste. Via an inflow from the nutrient reservoir, fresh nutrition is added to the culture vessel which usually contains a single or mixed bacterial population. Via an outflow to the tank, bacteria are harvested. Bacteria (*B*) grow in a chemostat feeding on a limiting resource *R*. The net growth rate of the bacteria is controlled by a constant dilution rate *D*. This is the rate of inflow and outflow from the chemostat. It represents the fraction of the volume in the chemostat being replaced per unit time.

Recently, Jeeves *et al.* [24] interrogated populations of *M. tuberculosis* cells cultured under fast dilution rates (0.03 h^−1^) or slow dilution rates (0.01 h^−1^) in continuous cultures (to achieve pre-drug exposure mean generation times of 23.1 h and 69.3 h, respectively) in order to understand how the slow growth of *M. tuberculosis* contributes to INH tolerance. Genotypic analyses were performed to determine the effect of different growth rates on the mutant frequency and the development of *katG* mutations. Phenotypic adaptation to INH under different growth rates was also explored using RNA tiling arrays. They found that bacterial numbers recover at both dilution rates after an initial rapid decline in population size. Under a fast dilution rate (0.03 h^−1^), numbers stabilized well below the original cell density prior to INH exposure, but under the slow dilution rate (0.01 h^−1^), the cultures were able to completely regain their original population size. The mechanisms permitting this complete recovery under the slow dilution rate but not under the fast dilution rate are not known. In addition, sequencing of the *katG* Ser315 locus of INH-resistant colonies isolated from the chemostat cultures (by plating on to 2× MIC INH) revealed distinct differences in the mutation profiles: under the slow dilution rate there were high frequencies of the *katG* codon Ser315 mutation (up to 37% of mutant colonies), whereas under fast dilution less than 14% of mutant colonies had a mutation at this locus. Rather, the colonies isolated from fast-dilution rates had a more diverse range of mutations elsewhere in the *katG* gene.

Here we use a simple mathematical model to explain the differences in the responses to INH of bacilli cultured at two different growth rates. We use a Markov chain Monte Carlo (MCMC) method to obtain posterior distributions of model parameters consistent with data. The results explain how the dilution rate affects the population dynamics. We use these posterior parameter distributions to define diverse collections of resistant subpopulations consistent with the observed growth curves, and place these resistant populations in a multi-population model to study diversity through time. We can account for the observed differences in population dynamics and diversity in a very simple way that does not require populations of slow-growing persisters, hypermutability or emergence of compensatory mutations (though these may be present nonetheless). We present testable predictions about how diversity is maintained in such cultures.

## 2. Material and methods

### 2.1. Experimental methods

The methods used to culture *M. tuberculosis* at different growth rates have been described previously (Jeeves *et al*. [24]). However, for ease of interpretation of the current study, the method is briefly described here. *Mycobacterium tuberculosis* (strain H37Rv) was grown in chemostats under controlled conditions as described previously [25]. We cultured *M. tuberculosis* on glycerol as the limiting nutrient. Three replicate cultures were performed at two different dilution rates to steady state [20,22]. The cultures achieved a mean generation time (MGT) of 23.1 h (dilution rate of 0.03 h^−1^; fast growth) or an MGT of 69.3 h (dilution rate 0.01 h^−1^; slow growth). The antibiotic was then added during steady state at a minimum inhibitory concentration (MIC) of 0.5 mg l^−1^ and maintained at this level in culture throughout each time-course. Viable count analyses were performed throughout the culture time-courses. In the main text, we present model analysis based on pooled replicates and in the electronic supplementary material we analyse replicate cultures separately.

### 2.2. Mathematical model

We developed a mathematical model for the continuous culture of *M. tuberculosis* cells exposed to MIC levels of INH. In the basic model, we include two bacterial subpopulations: *B*_1_, sensitive to INH and *B*_2_, resistant to INH. Resistant bacilli arise from the sensitive population by mutation at rate *μ* (we do not consider reverse mutation). Both subpopulations grow by consumption of the resources and are washed out at the (constant) dilution rate. The resources are consumed by the bacteria at a rate proportional to their resource-dependent growth rate and a conversion efficiency parameter *ε*, which is the amount of resource required to produce a single new cell [26].

The sensitive and resistant subpopulations have different maximum growth rates (*λ_i_*), different yield constants (*ε_i_*) and different half saturation constants (*k_i_*; the concentration of resource at which a population reaches half its maximum growth rate), so resistance can confer a fitness cost. The resource-dependent growth rate, *ϕ_i_*(*R*) for the two populations, is given by 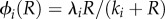. Models of continuous culture are well established and typically follow this pattern [27–29].

We model the effect of INH using two parameters, 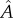 and *P*, to represent reduction in growth (by a factor 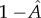) and bactericidal effect (at rate *P*), respectively. We also consider a situation where drug efficacy depends on growth rate and where bacteria whose growth is suppressed by the drug consume less of the resource rather than simply producing less growth for a given resource consumption (see the electronic supplementary material); results are unchanged by these modifications.

The model equations are

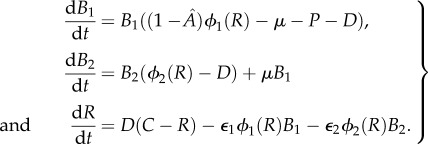


For a full description of the parameters, see table 1.

**Table 1.** Definitions of all states and parameters.

symbol	definition		
states			
*B*_1_	susceptible bacterial subpopulation (CFU ml^−1^)		
*B*_2_	resistant subpopulation (CFU ml^−1^)		
*R*	resource concentration (mg ml^−1^)		
variables		priors ∼ *U*(*a*, *b*)	
		*a*	*b*
*μ*	mutation rate (h^−1^)	2.56 × 10^−8^	2 × 10^−6^
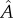	bacteriostatic action of INH	0.01	0.99
*P*	antibiotic bactericidal rate (h^−1^)	0	4
*ε*_1_	conversion efficiency parameter of *B*_1_ (mg CFU^−1^)	5 × 10^−10^	2 × 10^−8^
*ε*_2_	conversion efficiency parameter of *B*_2_ (mg CFU^−1^)	5 × 10^−9^	2 × 10^−7^
*D*	dilution rate (h^−1^)	—	—
*C*	resource input concentration (mg l^−1^)	—	—
*λ*_1_	maximum growth rate of susceptible subpopulation (h^−1^)	0.03	0.05
*λ*_2_	maximum growth rate of resistant subpopulation (h^−1^)	0.02	0.04
*k*_1_	half saturation constant of susceptible subpopulation (mg l^−1^)	0.01	0.03
*k*_2_	half saturation constant of resistant subpopulation (mg l^−1^)	0.02	0.06

### 2.3. Model parameters and Bayesian Markov Chain Monte Carlo approach

We used a Bayesian MCMC approach to find posterior sets of parameters related to antibiotic activity (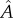, *P*), half saturation constant (*k*), conversion efficiency parameter (*ε*) and mutation rate (*μ*). We fit to all data points (replicate cultures) pooled together; fits to individual cultures are provided in the electronic supplementary material. *Mycobacterium tuberculosis* cells are generally characterized as being slow growers with a maximum growth rate not exceeding 1/16 divisions per hour in optimum conditions [19]. We choose values of *λ*_m_ within the range (1/23−1/20) h^−1^ [30]. For the fitting procedure, the smallest mutation rate of *M. tuberculosis* cells during exposure to INH is chosen to be 2.56 × 10^−8^ per cell per generation [31]. We do not include the dilution rate *D* and nutrient concentration *C* in the MCMC procedure as these are known and fixed.

Prior to the addition of INH to the chemostat cultures, the *M. tuberculosis* cells were grown to steady state at densities of approximately 10^8^ CFU ml^−1^. During this period of growth, mutation can occur, and it can be expected that small numbers of different subpopulations will arise from these large populations undergoing continuous turnover [32]. Accordingly, it is very unlikely that there would be absolutely no cells of minority subpopulations in a system with 10^8^ CFU ml^−1^. Despite this, we avoid directly introducing resistant subpopulations, and instead initialize the model with sensitive cells only, at their steady-state values prior to addition of INH. We allow the resistant subpopulation to arise through mutation and persist (or not, depending on its fitness in the system without INH) to reach its steady state before we add INH. We also set the initial value of *R* to be the pre-drug steady-state value.

We used MCMC to fit model parameters by maximizing the likelihood derived from the assumption of Gaussian scatter (minimizing the sum of squared differences), using the Matlab MCMC package by Haario *et al.* [33]. We based our likelihood on the weighted squared error function *χ*^2^(*θ*) in the usual way, with

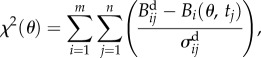
where 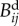 are the *n* data points for each observable *m*, *B*(*θ*, *t_i_*) is the solution of the *m*-dimensional dynamical system at time point *i*, and 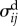 are the corresponding measurement errors [34]. This is equivalent to using the likelihood *L*(*θ*) [35], where
2.1
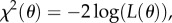
under the assumption of Gaussian noise. The MCMC procedure produces a posterior collection of parameter sets corresponding to model trajectories that fit the data.

### 2.4. Multi-population model

The posterior collections of parameters represent alternative parameters under which the model can match the observed data. As is often the case with dynamical models of biological systems, parameters are not uniquely determined by observations, due to complex nonlinear dependencies and trade-offs. We take the posterior to represent diverse ways that we can define a model subpopulation that is tolerant of INH and whose population dynamics follow the observed data. Accordingly, we use the posterior to extend the model to represent multiple, different subpopulations. We model *m* distinct mutations that could cause an *M. tuberculosis* bacillus to evade the action of INH. The susceptible subpopulation *B*_1_ mutates to create each of these *m* resistant subpopulations at different rates. Each subpopulation takes its parameter combination (*λ*_2_, *ε*_2_, *k*_2_) from the estimated posterior, so that each subpopulation exhibits different maximum growth rates *λ_i_*(*R*), different yield constants *ε_i_* and different half saturation constants, *k_i_*, as we assume that some mutations are more expensive than others. This allows us to use the posterior parameter distributions for resistant subpopulation to explore the relationship between mutation and selection (which has multiple flavours: drug resistance/tolerance, growth before dilution and efficient use of resources) in this system.

The differential equations are
2.2
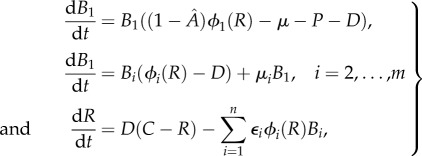
where

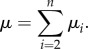


We assume that mutations giving rise to the different subpopulations occur at different rates, i.e. we use different values of *μ_i_*. If the subpopulation with the least fitness costs are assumed to appear faster than other subpopulations, then this gives them an advantage. Therefore, in our model simulation, we assume that mutations that confer higher net growth occur at a lower rate than mutations with lower net growth. Removing this assumption does not change the population dynamics, likely because the ultimate fate of each subpopulation depends more on its fitness cost than on the rate at which it appears in the system.

### 2.5. Diversity measure

A range of diversity indices have been used with bacterial communities [36–38]. Here we analyse diversity using Shannon's index *H*′ [39], which is defined as 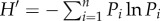, where *P_i_* is the proportion of the total population occupied by subpopulation *i*.

## 3. Results

**Figure 1.**
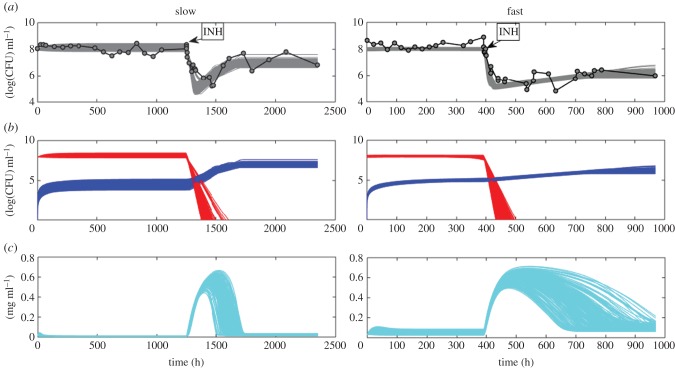
Grey lines (*a*) show the fits from 500 parameter sets randomly drawn from the posterior distribution of viable bacterial numbers for slow and fast dilution rates, the red lines (*b*) are the *B*_1_ population (INH sensitive), the blue lines are the *B*_2_ population (INH resistant) and the cyan (*c*) lines are plots of the resource concentration. (Bacterial counts are in log base 10). Right panels: long transient dynamics have been suppressed.

The model captures the qualitative behaviour of the cultures in [24] under fast- and slow dilution rates. No fine tuning of parameters is necessary: the model is robust in the sense that a range of parameters fits the data as shown in figure 1. For baseline values of parameter fits for slow and fast dilution rates, see tables 2 and 3, respectively. Matching the observed data does not require modelling slow-growing persisters or an explicit lag phase. However, the mutation rates and large population sizes result in a sizable minority population (of order 10^5^) of resistant bacteria at the time that INH is added; this is a combination of inferred mutation rates and growth and turnover of the steady-state population of order 10^8^ or higher prior to the addition of INH. We also note that in order to fit the steady-state bacterial numbers before the addition of INH, we require differences in the yield coefficient *ε_i_* between the two dilution rates. While this appears counterintuitive, it has been predicted previously [19,40,41], and may also be related to the higher expression of genes involved in lipid metabolism and ATP synthesis under fast dilution post-INH addition [24]. Pressure to use the carbon source most efficiently may be weaker under fast dilution than slow dilution, consistent with the model's prediction of excess glycerol in the medium.

**Table 2.** Baseline values of parameter fits (slow dilution).

parameter	mean	2.5th percentile	97.5th percentile
*μ*	3.01 × 10^−6^	2.32 × 10^−6^	6.68 × 10^−6^
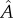	0.04	0.027	0.95
*P*	0.06	0.03	0.10
*ε*_1_	5.46 × 10^−8^	2.61 × 10^−9^	1.07 × 10^−8^
*ε*_2_	8.46 × 10^−8^	2.69 × 10^−8^	1.77 × 10^−7^
*λ*_1_	0.041	0.031	0.049
*λ*_2_	0.027	0.021	0.037
*k*_1_	0.019	0.01	0.03
*k*_2_	0.04	0.02	0.05

**Table 3.** Baseline values of parameter fits (fast dilution).

parameter	mean	2.5th percentile	97.5th percentile
*μ*	5.33 × 10^−6^	2.14 × 10^−6^	6.94 × 10^−6^
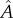	0.50	0.03	0.96
*P*	0.06	0.03	0.09
*ε*_1_	7.29 × 10^−9^	5.06 × 10^−9^	1.26 × 10^−8^
*ε*_2_	4.83 × 10^−7^	1.07 × 10^−7^	1.08 × 10^−6^
*λ*_1_	0.03	0.04	0.05
*λ*_2_	0.03	0.036	0.04
*k*_1_	0.02	0.01	0.03
*k*_2_	0.03	0.02	0.04

Figure 1*a* shows the model's growth curves alongside observed data. The susceptible population *B*_1_ dies off rapidly upon addition of INH, leading to a temporary excess of glycerol. Figure 1*b* shows that the resistant subpopulation begins to grow immediately—the apparent lag phase in the rise of this resistant population is due to the initial decline in the total viable count because the vast majority of bacteria are sensitive to INH (type *B*_1_ in the model) when the antibiotic is first added. While the population size begins to expand again in both the fast- and slow dilution rates, under slow dilution the bacteria have time to maximize use of the excess nutrient with an apparent increase in growth rate, which in turn enables the population to re-establish the original population size. By contrast, a lower but fairly constant population size of resistant bacteria is eventually maintained under the fast dilution rate (as observed by Jeeves *et al*. [24]). To aid interpretation of this, at the time that INH was added to the cultures, the bacteria under fast dilution were dividing at a rate very close to the maximum physiologically achievable growth rate for *M. tuberculosis*, as it has been determined previously that increasing the dilution rate to higher than a MGT of 23.1 h MGT resulted in wash-out of the cultures. If this is the maximum physiologically achievable growth rate, then an increase in the growth rate (and, therefore, an increase in biomass) is not possible even in the presence of excess glycerol. This may account for why fast dilution cultures could not recover their original population size. We note, however, that if left to run for a longer time period, the cultures under fast dilution may very slowly increase, possibly up to similar levels to those attained by the cultures under slow dilution.

**Figure 2.**
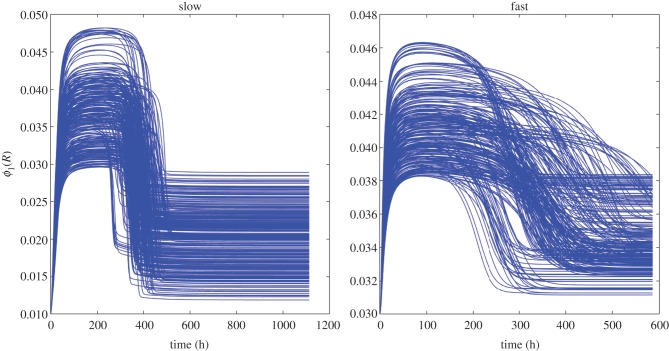
Plots of the resource-dependent growth rate *ϕ_i_*(*R*) for fast and slow dilution rates over time. Note that growth is frequently greater than the dilution rate due to the departure from steady-state conditions. (Online version in colour.)

Under steady-state conditions, the imposed slow and fast dilution rates (0.01 h^−1^ and 0.03 h^−1^, respectively) in the chemostat correspond to slow and fast bacterial growth, and this is a motivation for the use of continuous cultures as models for chronic disease (slow dilution rates) and acute disease (fast dilution rates) *M. tuberculosis* infection [22,42] (tables 2 and 3). The comparison of two distinctly different growth rates in a controlled and defined system enabled us to measure the direct effect of growth rate on the response of *M. tuberculosis* to INH exposure. However, once the system departs from steady state (in this case when INH was added), growth rates and dilution rates are not necessarily the same. At times, the growth rate under slow dilution reached the growth rates imposed by fast dilution (figure 2).

**Figure 3.**
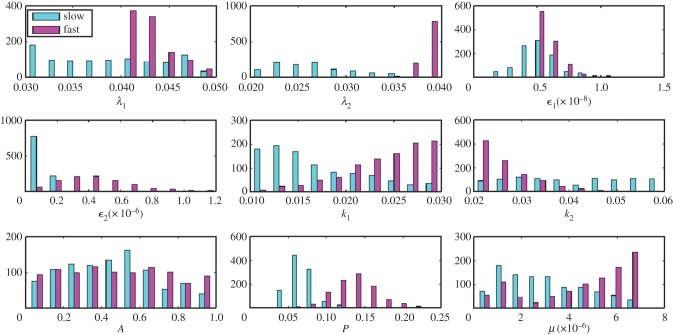
Histograms of 1000 random samples of posterior parameter distributions for slow and fast dilution rates.

There are wide ranges of posterior parameters that allow the model the match the data, and most posterior parameters are similar under fast and slow dilution (figure 3). A few show marked differences: *λ*_2_, *k*_1_, *P*, *μ* and *ε*_2_ are higher under fast dilution and there are some correlations (electronic supplementary material, figures S4, S5). The kill rate *P* and mutation rate *μ* have a difference of about an order of magnitude between the two dilution rates, consistent with Jeeves *et al*.'s measurement of mutation rates of 10^−7^ and 10^−6^ per cell per generation in slow- and fast dilution, respectively. We recognize that the model has parameters whose roles overlap: for example, both 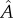 and *P* reflect antibiotic action, and both *λ_i_* and *k_i_* determine how growth depends on the resource. As such, parameters are not statistically identifiable from the data we have, and instead of attempting to find maximum-likelihood estimates of each parameter we have taken a Bayesian approach to find posterior collections of parameters sets where the model matches the data. We draw conclusions not about the values of specific parameters, but about dynamics we find using *all* posterior sets of parameters. We explore further identifiability and correlation structure in the posteriors in the electronic supplementary material.

The posterior parameters defining the resistant subpopulation allow us to explore the estimated fitness cost of INH resistance. An intuitive way to define the relative fitness in this context is the ratio of growth rates between resistant and sensitive subpopulations:

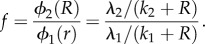


**Figure 4.**
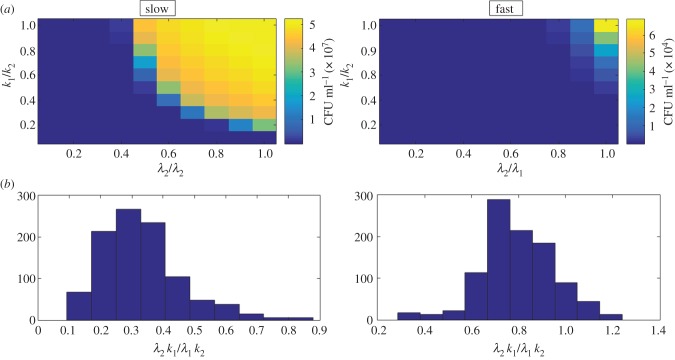
Heatmaps (*a*) showing how culture recovery depends on fitness costs and histograms (*b*) of relative fitness 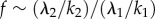 of 1000 posterior parameter sets. Recovery strength ranges from dark blue (weakest recovery) to bright yellow (strongest recovery).

This depends on the level of resource *R*: relative fitness is specific to an environment. When 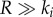, 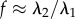, and when 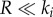, 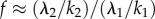. Figure 4 shows how the recovery is affected by *λ*_2_ and *k*_2_, under slow- and fast dilution rates. Under fast dilution, the recovery depends very sensitively on the fitness costs, whereas under slow dilution, recovery is much more robust to small changes in the growth parameters. The lower panels of figure 4 shows the posterior relative fitness values 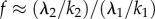 for the two dilution rates. While slow dilution allowed more rapid recovery of the bacterial population, the relative fitness values of the minority subpopulation that are performing this recovery are notably lower than the corresponding fast dilution subpopulation. This is because under slow dilution, subpopulations can stay in the system long enough to consume the resource; competition for this now-depleted resource means that the most fit quickly dominates. Under fast dilution, all remaining subpopulations can grow quickly enough to outpace the rapid dilution, but they cannot consume all of the resource before being washed out (they do eventually, but not until quite a bit later than their slow dilution counterparts).

**Figure 5.**
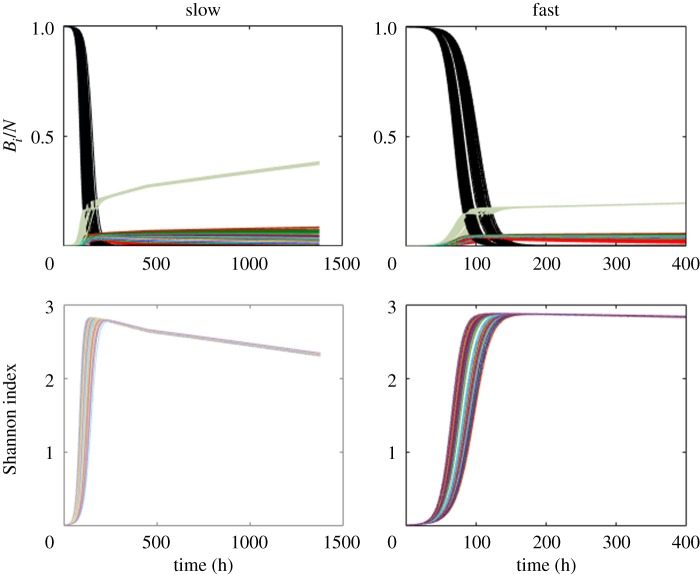
Proportion plots and plots of the Shannon diversity index *H*′ for 1000 parameter sets of the multi-population model: with 20 resistant subpopulations competing after the susceptible subpopulation (black) dies off. The fittest subpopulation (green) maintains the highest proportion under both dilution rates. (Online version in colour.)

We explored a model with a diverse resistant subpopulations whose parameters are drawn from the posterior distribution that matches the data and found that the diversity patterns differ markedly under the two dilution rates (figure 5), with fast dilution allowing a wider diversity of subpopulations for a much longer period than slow dilution. Previously, mutants were isolated from fast and slow dilution rates by selection on agar containing 2× MIC INH, and resistance was characterized by sequence analysis of katG, which encodes the catalase-peroxidase activity and is responsible for activation of INH. In parallel with our observations in the model, greater diversity in the katG gene was observed under a fast dilution than slow [24]. This may be surprising given that fast dilution provides very strong selective pressure for rapid division. However, the model predicts that there is an excess of the growth resource under fast dilution, with the effect that there is reduced competition for resources between subpopulations. The effect of dilution rate on diverse population interactions in chemostat systems has been noted previously [43,44], without the focus on resistance and diversity.

In chemostat cultures of multiple competing populations, it is known that the subpopulation with the smallest break-even resource concentration wins and competitively excludes the others [29,45,46]. A population's break-even concentration is the resource concentration it requires to support growth that balances death and dilution. It depends on the dilution rate and two parameters intrinsic to the population, *λ_i_* and *k_i_*, which we draw from posterior parameter values to reflect different fitness costs. After the susceptible bacteria have been depleted, the system, therefore, reduces to one with several competing subpopulations that are unaffected by antibiotic action (all are resistant). The fitter a population is, the lower its break-even concentration. The proportion of the total population occupied by a subpopulation varies inversely with its fitness cost. After the susceptible subpopulation dies off, the resistant subpopulation with the least fitness cost will have the highest proportion of the total population (figure 5). Accordingly, in our fast dilution model system, the model cultures will eventually reach a steady state with one dominant subpopulation (ignoring onward generation of diversity by mutation), but our simulations indicate that it would take very long times to reach this equilibrium. We did not model the mechanisms of INH resistance and their dependence on the dilution rate, but it has been observed that susceptibility to ribosome-targeting antibiotics can be decreased under slow growth [47] in other organisms. Here, while growth parallels dilution rate at steady state, it is not fixed during the experiment. Furthermore, the observed recovery of the bacterial populations reflects not only the strength of INH resistance but also the resource availability, competition and dilution.

## 4. Discussion

We modelled *M. tuberculosis* growth and diversity in continuous culture under the action of INH. We obtained posterior parameter distributions for which the model matches observed data on the recovery of the population during addition of INH, and explored the dynamics of diverse INH-resistant subpopulations consistent with these data. The model robustly matches observed growth curves and provides a platform for exploring the emergence of resistance to INH in continuous culture. While the posteriors contain numerous sets of model parameters that allow the model to match observations, all of these parameter sets give rise to the conclusions we report. Even though fast dilution provides very strong selective pressure in favour of rapid cell division, fast dilution resulted in excess resource compared to slow dilution, and the diversity of model subpopulations that could be maintained over long periods was greater under fast dilution. This is consistent with the observations of Jeeves *et al*. [24] regarding diversity in *katG* mutations. We interpret this result in the context of fitness and inter-population competition.

Mutation-selection dynamics lead to removal of non-beneficial mutations in a population. However, in a system like ours where there are several beneficial mutations which vary in fitness, the balance between mutation and selection is complex as selection acts in several distinct ways on this variation [48]. Fitness is often framed as a one-dimensional variable capturing an organism's overall capability for growth. This depends on the environment as well as on the organism itself. In our continuous culture system, the ability to divide before being washed out, the strength of resistance to INH, and the ability to consume the carbon source before competitors and to use the carbon efficiently are distinct elements of the fitness of subpopulations. The relative importance of these aspects of fitness is different under the two dilution rates. Fast dilution confers strong selection in favour of rapid division, and might, on that basis, be expected to permit less diversity than slow dilution. However, fast dilution also results in excess glycerol in the medium, weakening competition for resources. It appears that the effects of reduced competition are a stronger driver of diversity in this system than the effects of strong selection for rapid growth. The fact that the conversion parameter *ε*_2_ was inferred to be much larger under fast dilution than under slow dilution supports the notion that selection for rapid carbon usage is lower under fast dilution.

An increased capacity of cultures to give rise to mutants (reflected in the mutation rate) increases the adaptability of these cultures to harsh conditions [49]; this could be reflective of the adaptation of clinical populations to the host environment. As the posterior values of the mutation rate are notably higher under fast dilution than under slow dilution, one might expect that cultures in fast dilution would be able to recover more easily. However, the potential advantages conferred by rapid mutation may be outweighed by the pressure of fast dilution, the occurrence of deleterious mutations and the fact that the fast dilution rate may reach the physiological limit of *M. tuberculosis*' growth capacity. All these factors combine to affect the outcome of mutation-selection balance in this system, resulting in higher long-term diversity under fast dilution than under slow dilution.

The competitive exclusion principle observed in chemostat cultures is based on the assumption that the cultures do not undergo continuous mutations. However, this is not the situation when a clonal population diversifies into a number of subpopulations [49–51]. Continuous mutation maintains long-term diversity with many minority subpopulations, and would do so under either dilution rate. We do not explicitly model diversity arising from resistant subpopulation, instead assuming that each subpopulation's descendants carry the subpopulation's growth parameters. The diversity we model, therefore, reflects the diversity of phenotypic growth parameters rather than overall genetic diversity, which would continue to accrue at a low rate consistent with the low genome-wide mutation rate of *M. tuberculosis*.

The model raises questions that can be answered in further chemostat studies: does high resource availability delay the end course of exploitative competition? What determines the mutation rate and how does it affect the evolution and fitness of resistance? Studies combining whole-genome sequencing with monitoring of the resource concentration will provide tests of the predictions we have made, as well as insights as to the continuous generation of diversity after the drug-sensitive cells have died out.

The emergence of fit DR TB subpopulations is a major concern for global health. Gagneux *et al.* [11] report that mutant subpopulations with the least fitness cost to rifampicin are the most prevalent among rifampicin-resistant isolates. Many tuberculosis patients are hosts to resistant subpopulations arising during growth and turnover of drug-sensitive infections [11,52–54]. Following early studies that found that TB patients given a single drug developed resistance due to minority subpopulations [25,55], standard TB treatment regimens include four antibiotics taken over many months. Resistant minority populations have a selective advantage in patients during drug therapy and may spread onwards, gaining in fitness over time. It is reasonable to assume that upon emergence, resistant subpopulations compete with their drug-susceptible progenitors for resources. If continuous culture systems are a model for in-host infection, our results suggest that drug selection occurs when host resources are likely to be plentiful—during slow growth periods of the infection or when bacterial numbers have declined after early bactericidal action—giving rise to diverse resistant subpopulations. Our model does not require slow-growing persisters or hypermutability in response to antibiotic action or other advantages beyond simple mutation in order to match observed complete recovery of bacterial populations. Even in the very simple, constrained and highly selective environment of nutrient-limited continuous culture systems, *M. tuberculosis* can rapidly generate fit DR mutants that can establish long-term survival.

## Supplementary Material

Supplementary material
