# Supplementary material for: Diverse drug-resistant subpopulations of Mycobacterium tuberculosis are sustained in continuous culture
Source: J R Soc Interface. 2016 Nov;13(124):20160745. doi: 10.1098/rsif.2016.0745 (PMC5134024; doi:10.1098/rsif.2016.0745)
Supplement: Supplementary material [file rsif20160745supp1.pdf]

Supplementary material

1 Fits to individual cultures

Three replicate cultures were run concurrently for both fast and slow dilution rates. As a result, there are three datasets to which we fit the data (Figure 1).

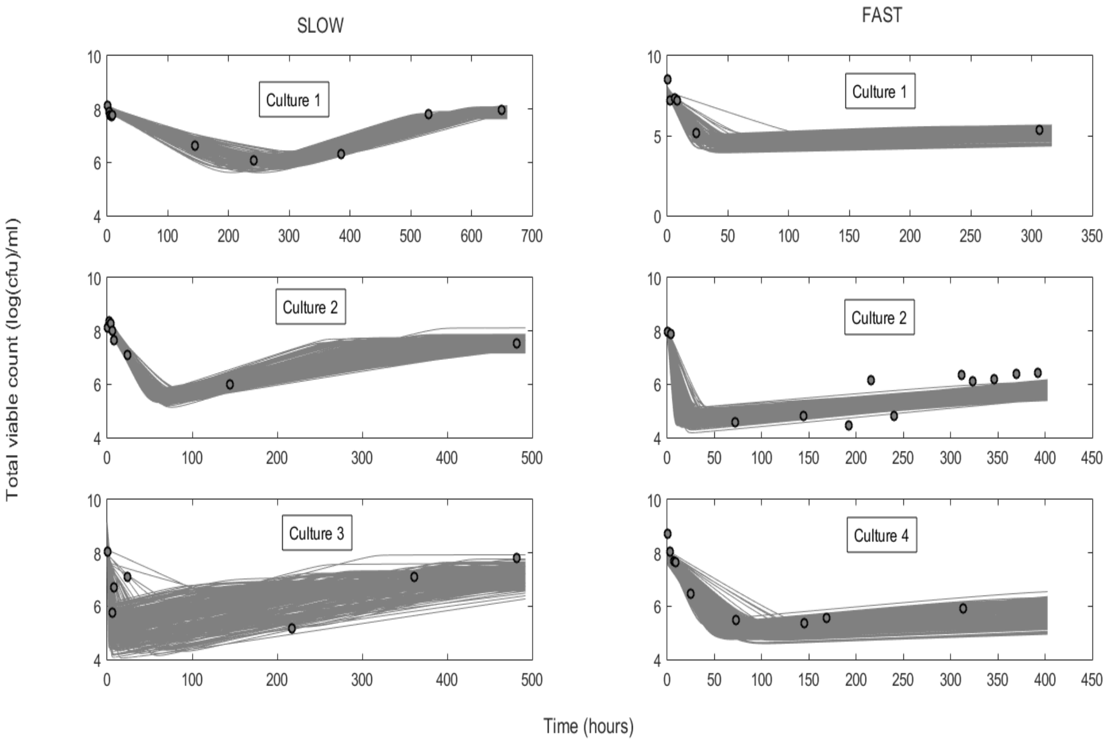

Figure 1: Grey lines show the fits from 500 parameter sets randomly drawn from the posterior distribution resulting from running the MCMC seperately on the individual replicated cultures

Figure 2 shows that most of the posterior parameter values are similar across the individual cultures for each dilution rate. As was observed in the fits to combined replicates, the values of P are higher under slow dilution rates. However, some of the patterns observed in the combined fits (i.e. *epsilon* and μ greater under fast than slow dilution) are not observed in the individual fits. This may be as a result of the fact that the numbers of data points for individual cultures are not sufficient to capture these trends.

2 Model with action of INH depending on growth rate

Drugs that target the metabolic process are likely to be less effective against metabolically inactive bacteria than fast-growing bacteria ([1],[6]). In the modelling context this motivates allowing drug efficacy to change with growth rate [2]. We compared our results from the main text to results from a model designed to accommodate this, in which the bactericidal action as well as the growth inhibition increases as the growth rate increases. In addition, in the main text our model assumption was that the action of INH did not affect the rate at which bacteria consume the resource; rather, resource was consumed at the same rate but the growth achieved was reduced. In contrast, here we explore the assumption that as INH reduces growth, less resource is consumed by the susceptible population. With these two modifications, the model becomes:

$$\frac{dB_1}{dt} = B_1((1-\hat{A})\phi_1(R) - \mu - P(1+\frac{R}{k_1+R}) - D),$$
$$\frac{dB_2}{dt} = B_2(\phi_2(R) - D) + \mu B_1,$$
$$\frac{dR}{dt} = D(C-R) - \epsilon_1\phi_1(R)(1-\hat{A})B_1 - \epsilon_2\phi_2(R)B_2.$$

The fits to this model are shown in Figure 3, and Figure 4 shows the diversity patterns under the same simulation approach as in the main text. Results are consistent: we obtain increased diversity for long periods under fast dilution as in the model presented in the main text.

3 Parameter identifiability

When a model is fitted to experimental data, the question of how reliable parameter estimates are arises. Strong correlations and functional relationships between parameters and may prevent them from being uniquely identified. There may exist parameters which can vary several orders of magnitude without affecting the quality of fit. Our aim in this work is not to use the model to identify values of the parameters, but to find the origins of the recovery dynamics and diversity patterns observed in slow and fast dilution. For these purposes, a model whose fits to observation are not highly sensitive to individual values of unknown parameters is an advantage, as the model's structure robustly captures the observed dynamics. In contrast, for parameter estimation, such sensitivity would be desirable. Despite this difference in aims, in this section we explore parameter identifiabilty.

Figure 5 shows correlation plots of the parameters under slow and fast dilution rates; several parameter pairs are significantly correlated. Under both dilution rates, there is a negative correlation between $\hat{A}$ and P. This means that they are not independent, and indeed their effects are related (they model INH reducing bacterial numbers through growth inhibition and bactericidal action). The dependence of the parameters λ and k has been noted previously [4, 5]; both affect the growth rate.

Bayesian inference provides distributions of parameter values that are consistent with data. The Bayesian approach to fitting is well-suited to the situation where parameters are not identifiable; many combinations of parameters may suffice for the model to have a specified behaviour. Hines et al [3] provide a framework which uses the MCMC traces of Bayesian fits to check for identifiability. The structure of the posterior distribution reveals whether regions of very high posterior distribution

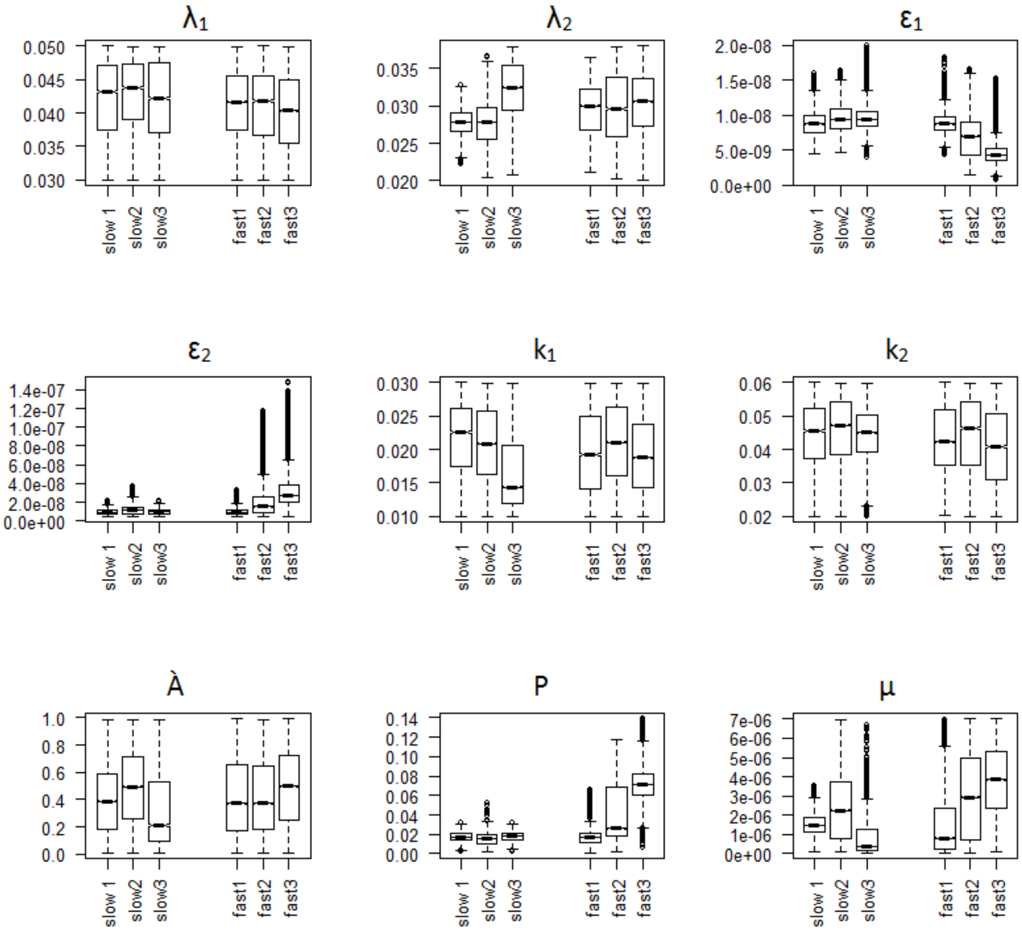

Figure 2: *Boxplots of 5000 random samples of posterior parameter distributions for slow and fast dilution rates (for individual cultures).*

are localized to specific regions in the parameter space. They propose that if the posterior parameter values in best agreement with the data are confined to a small region of the parameter space, then the model parameters are identifiable.

Figures 6 and 7 show density plots for selected pairs of parameters (those with correlation coefficient greater than 0.5; Figure 5). The densities of posterior parameter values have been used to generate maps with colours ranging from yellow to blue; areas of yellow shading correspond to higher posterior density. The joint posterior distributions of these model parameters indicate that the optimal estimates of some model parameters are contained in a small, bounded region of the parameter space, but this is not generally the case.

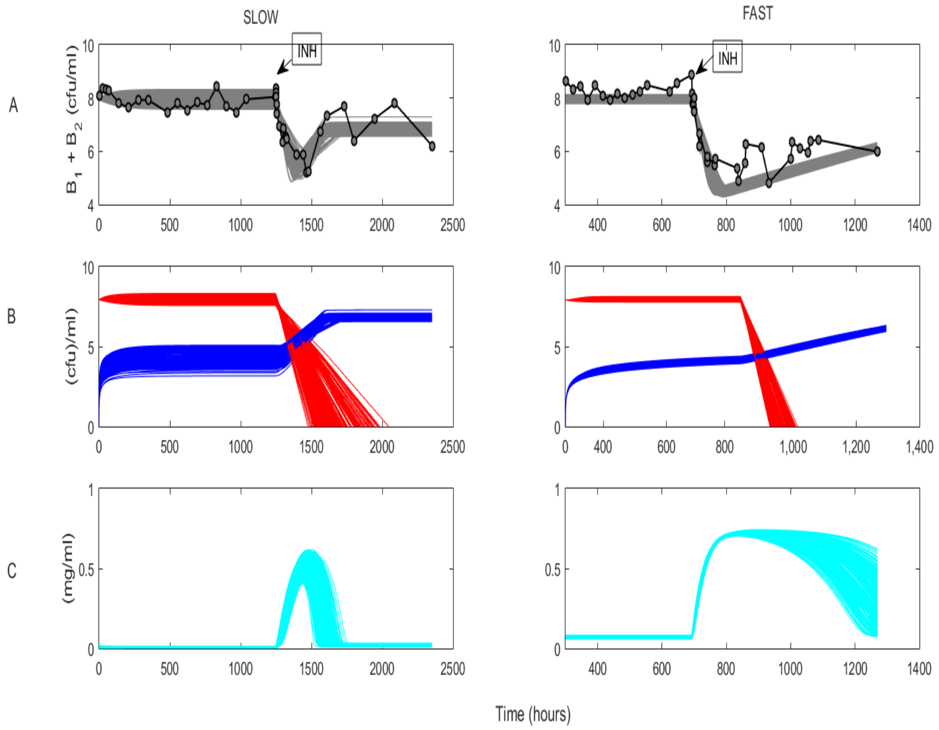

Figure 3: *Grey lines (A) show the fits from 500 parameter sets randomly drawn from the posterior distribution of viable bacterial numbers for slow and fast dilution rates, the red lines (B) are the B_1 population (INH sensitive), the blue lines are the B_2 population (INH resistant), and the cyan (C) lines are plots of the resource concentration.(bacterial counts are in log)*

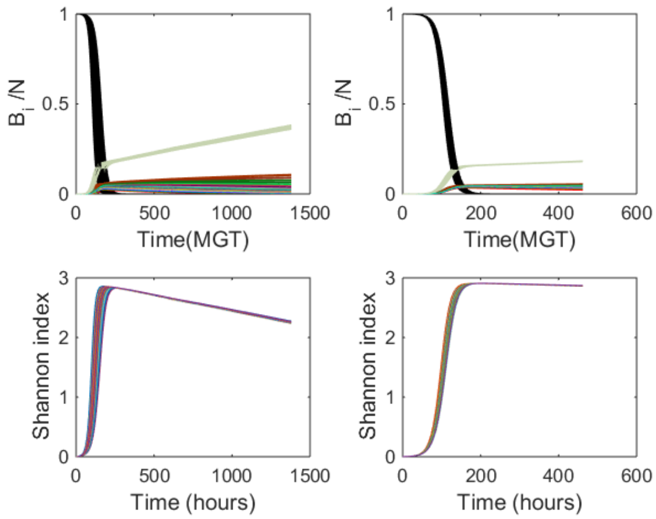

Figure 4: Diversity patterns in the modified model, reflecting the assumption that INH reduces the rate of resource consumption as well as the growth, and that its efficacy may change with growth rate. Resuls are consistent with those reported in the main text.

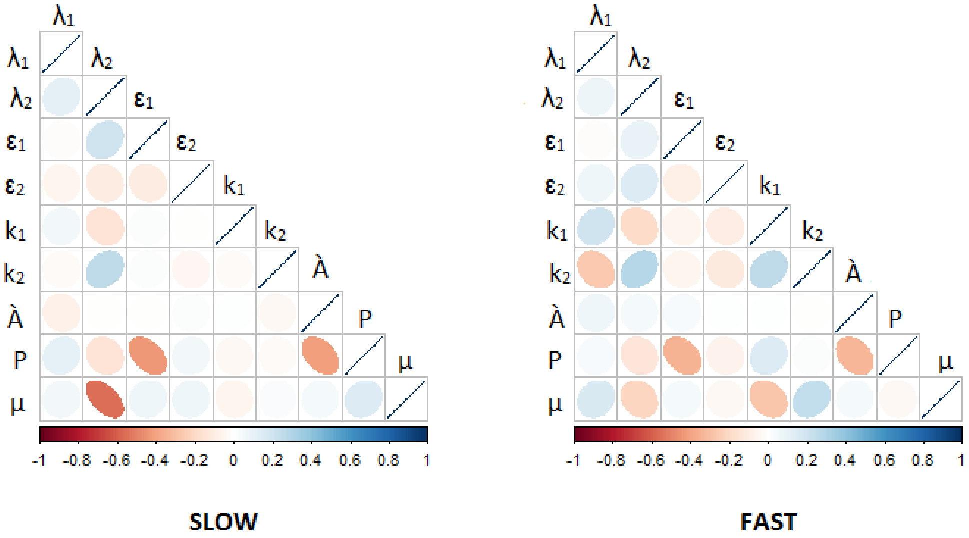

Figure 5: *Correlation plots of 1000 random samples of posterior parameter distributions for slow and fast dilution rates. Dark blue and dark red colors display parameters with high positive and negative linear dependencies, respectively*

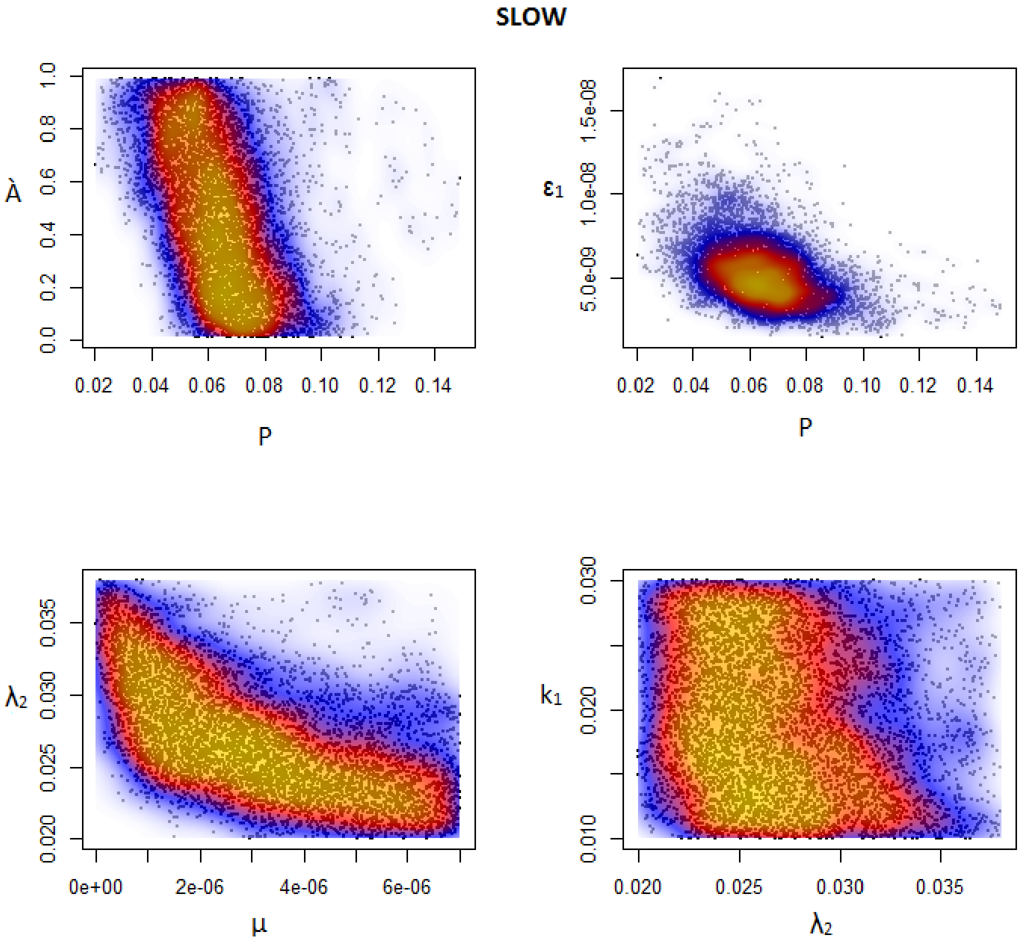

Figure 6: *Heat maps of the joint posterior distributions of parameters that are significantly correlated (with a correlation coefficient* $|r| \geq 0.5$*). Lighter, brighter areas correspond to areas of higher posterior density.*

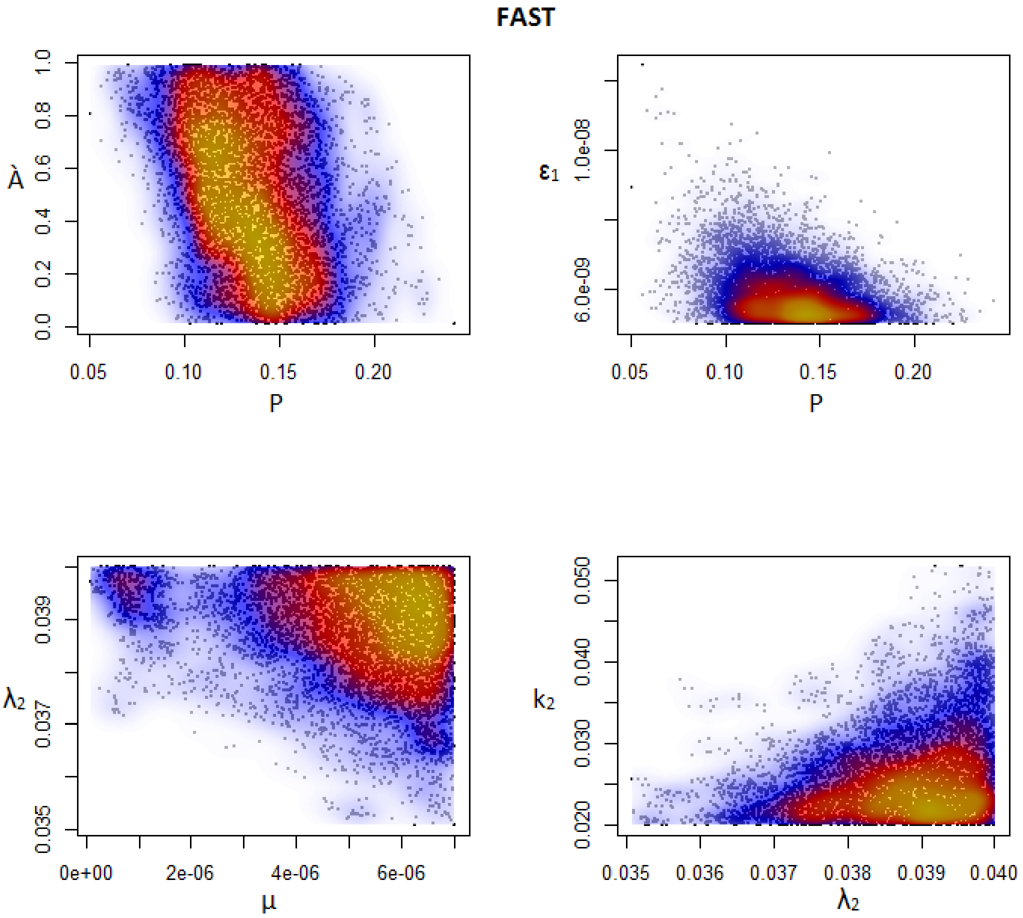

Figure 7: *Heat maps of the joint posterior distributions of parameters that are significantly correlated (with a correlation coefficient* $|r| \geq 0.5$*). Lighter, brighter areas correspond to areas of higher posterior density.*

4 References

[1] R. H. Eng, F. T. Padberg, S. M. Smith, E. N. Tan, and C. E. Cherubin. Bactericidal effects of antibiotics on slowly growing and nongrowing bacteria. *Antimicrobial agents and chemotherapy*, 35(9):1824–8, sep 1991.

[2] P. Greulich, M. Scott, M. R. Evans, and R. J. Allen. Growth-dependent bacterial susceptibility to ribosome-targeting antibiotics. *Molecular systems biology*, 11(1):796, jan 2015.

[3] K. E. Hines, T. R. Middendorf, and R. W. Aldrich. Determination of parameter identifiability in nonlinear biophysical models: A Bayesian approach. *The Journal of General Physiology*, 143(3):401–416, mar 2014.

[4] K. Kovárová-Kovar and T. Egli. Growth kinetics of suspended microbial cells: from single-substrate-controlled growth to mixed-substrate kinetics. *Microbiology and molecular biology reviews : MMBR*, 62(3):646–66, sep 1998.

[5] J. LOBRY, J. FLANDROIS, G. CARRET, and A. PAVE. Monod's bacterial growth model revisited. *Bulletin of Mathematical Biology*, 54(1):117–122, 1992.

[6] K. Poole. Bacterial stress responses as determinants of antimicrobial resistance. *Journal of Antimicrobial Chemotherapy*, 67(9):2069–2089, sep 2012.
